# Supplementary material for: Microbiome dynamics in tank- and pond-reared Genetically Improved Farmed Tilapia (GIFT)
Source: Front Microbiomes. 2025 Sep 1;4:1567816. doi: 10.3389/frmbi.2025.1567816 (PMC12993496; doi:10.3389/frmbi.2025.1567816)
Supplement: Supplementary Data Sheet 3 — PCA, coordinates of the barycenters of the PCA, and correlation and cos² values of Fish Age and Body Weight. [file DataSheet3.zip › Supplemental_Data3_PCA/Supplemental_Data3_Figure_PCA.pptx]

## Slide 1
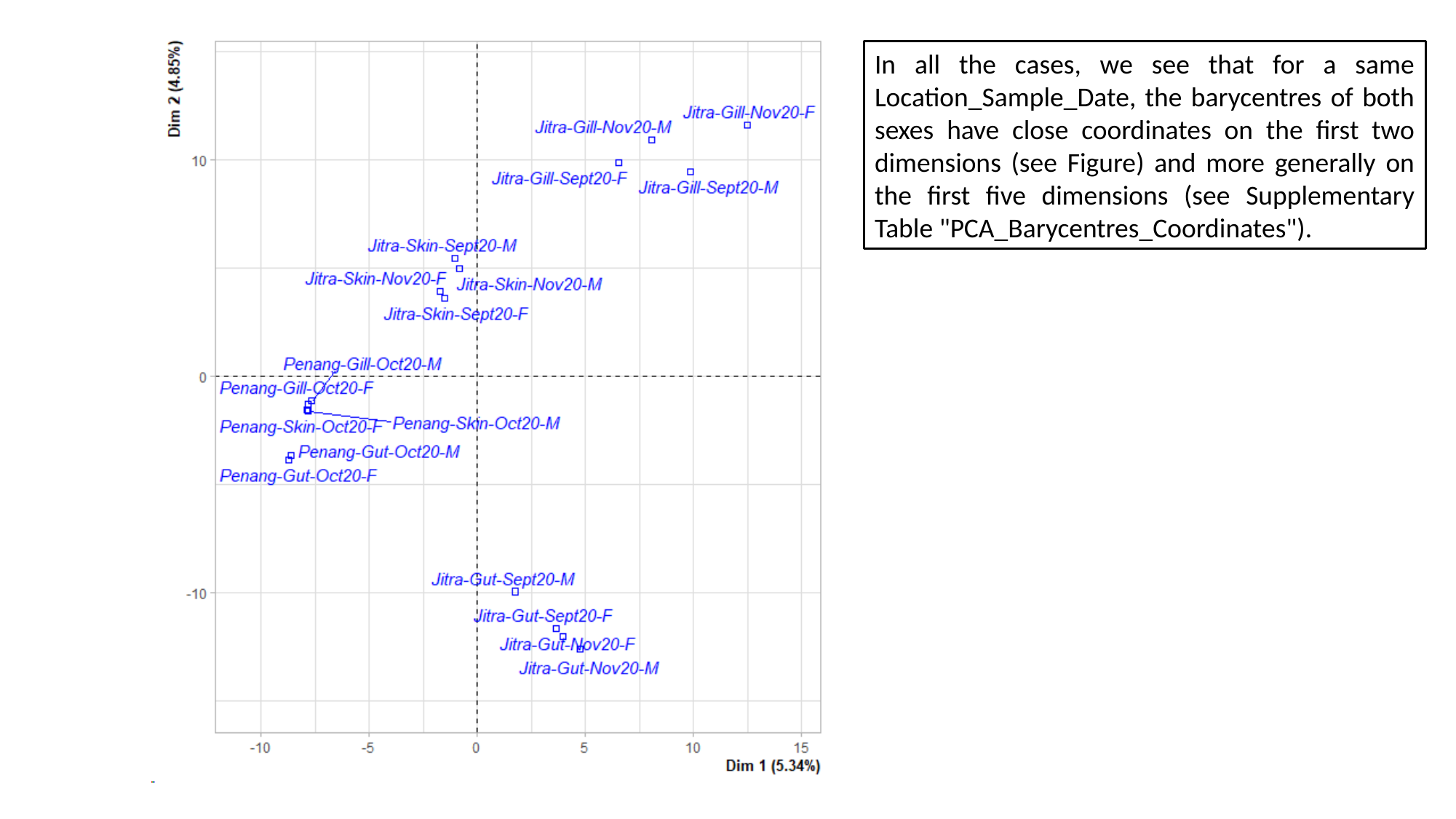

In all the cases, we see that for a same Location_Sample_Date, the barycentres of both sexes have close coordinates on the first two dimensions (see Figure) and more generally on the first five dimensions (see Supplementary Table "PCA_Barycentres_Coordinates").
